# 

**Published:** 1887-09-10

**Authors:** 


### CONTENTS.

**Sober Science** - - - - - - - 391
**Words of Consolation.**—Chapter XLIX. The Sufferings of Christ - - - - - - 392
**The Doctor's Pulpit.**—A Holiday Sermon - - 393
**Books for Hospitals.** By One of the Crowd - 394
**The Rod of Health.** By Bookworm - - - 395
**Amusements for Convalescents.** — Palmistry Notes. By a Lady - - - - - - 397
**Notes and News.**—Miscellaneous Items.—House-to-House Hospital Collections.—Open-air Concert in Aid of the Batley Cottage Hospital.—The Glasgow Medical Charities Committee.—Vacancies.—Game for Hospitals.—The Stratford-upon-Avon Infirmary.—Cheap Trips.—Annual Sermons.—Hospital Exhibition.—Workshop Collections.—Collecting Dogs.—The Scarlet Fever Epidemic.—The London Temperance Hospital.—Donations in Aid of the Building Fund of the Great Northern Central Hospital.—Mr. F. R. Russell on Scarlet Fever. 398
**Annotations.**—More Pasteur Failures.—Doctors and Druggists.—Anti-vaccinators - - - - - 401
**Kate Mountstephen.** By C. G. Furley.—Chap. III. A Hard Question - - - - - - - 402
**The National Pension Fund** - - - - 405
**The Editor's Letter-Box.**—A "Matron's Corner" - 405
**In- and Out-Patients' Hospital Diary.**—I.—General Hospitals - - - - - - - 406
**The Children's Corner.**—Puzzles, Answers, Letters, etc. - - - - - - - - vi
**Amusements with Profit** - - - - - vi

